# Computed Tomography Findings of Pulmonary* Mycobacterium simiae* Infection

**DOI:** 10.1155/2017/6913564

**Published:** 2017-01-03

**Authors:** Ayeh Baghizadeh, Payam Mehrian, Poopak Farnia

**Affiliations:** ^1^Pediatric Respiratory Diseases Research Center, National Research Institute of Tuberculosis and Lung Disease (NRITLD), Shahid Beheshti University of Medical Sciences, Tehran, Iran; ^2^Mycobacteriology Research Center, National Research Institute of Tuberculosis and Lung Disease (NRITLD), Shahid Beheshti University of Medical Sciences, Tehran, Iran; ^3^Department of Biotechnology, School of Advanced Technology in Medicine, Shahid Beheshti University of Medical Sciences, Tehran, Iran

## Abstract

Nontuberculous mycobacterial (NTM) pulmonary infections can be quite similar to tuberculosis, both clinically and radiologically. However, the treatment protocol is not similar.* Mycobacterium simiae* is a rare cause of NTM pulmonary infection. Herein, we aimed to evaluate and compare the computed tomography (CT) scan findings of* M. simiae* infection in lungs. For this reason, thirty-four patients (*n* = 34) with* M. simiae* lung infection were retrospectively evaluated. Diagnosis was confirmed by American Thoracic Society (ATS) guidelines and CT scans were reviewed in both lung and mediastinal windows. The average age of patients was 63 ± 14.54 years and 52.9% were male. The majority of patients had cough (91.2%) and sputum production (76.5%). Clinically, 41.2% of patients had previous history of TB (14/34), 38.2% had cardiac diseases (13/34), and 35.3% had diabetes mellitus (12/34). The most common CT findings in our study were nodular lesions (100%) and bronchiectasis (85.29%). Regarding the severity, grade I bronchiectasis was the most prevalent. Other prominent findings were tree-in-bud sign (88.2%), consolidation (52.94%), and lobar fibrosis and volume loss (67.6%). There was no significant zonal distribution of findings. In conclusion, nodular lesions and bronchiectasis are the most frequent features in CT scan of* M. simiae* pulmonary infection.

## 1. Introduction

Nontuberculous mycobacteria (NTM) are a large group of pathogens comprising more than 150 species and the number is continuously growing [1]. These opportunistic pathogens account for an increasing proportion of mycobacterial infections [2–4]. Reports indicate that the incidence and mortality rate of these infections have a rising trend [5, 6].* M. simiae* is one of the rare species of NTM, first described in 1965 [7]. Up to now,* M. simiae* has been reported from many regions in the world but the most prevalent regions have been Southern United States and Western Asian countries [8–10].

*M. simiae* can cause infections in various systems of body, including lungs.* M. simiae* pulmonary symptoms are nonspecific and include a combination of pulmonary and constitutional symptoms such as cough, sputum production, hemoptysis, dyspnea, fever, sweating, and weight loss [11, 12]. Underlying pulmonary diseases increase the risk of infection [12].* M. simiae* infection is more prevalent in immunocompromised patients, including HIV positive patients, patients with previous history of TB, children, and the elderly [13–16]. Diabetes mellitus, cardiovascular diseases, and malignancies also increase the risk of infection [14].

Chest X-ray findings in NTM infections are usually nonspecific. These findings often do not help distinguish these infections from other conditions such as TB [17–19]. There are very few studies regarding CT scan of pulmonary* M. simiae* infection. Most of them have been conducted in AIDS or other immunocompromised patients and failed to reveal any specific findings [14, 20]. In this study, we evaluated CT scan findings of pulmonary* M. simiae* infection.

## 2. Methods and Materials

Between January 2011 and January 2016, 34 patients with* M. simiae* lung disease referred to Masih Daneshvari Hospital were retrospectively evaluated. In all subjects, the diagnosis of mycobacterial infection was confirmed by Masih Daneshvari Microbiology Laboratory with respect to the guidelines of the ATS, considering related symptoms, consistent radiographic abnormalities, and culture-positive sputum specimens for making a diagnosis [7]. Patients with high clinical suspicion for tuberculosis who had negative sputum cultures underwent bronchoscopy with bronchoalveolar lavage and transbronchial biopsy for a definite diagnosis. All patients underwent pulmonary CT scan. The demographic data were obtained from medical records.

CT scan was acquired with a helical technique from the lung apices to the lung bases. The images were reviewed on both lung (window width 1500 H, window level −700 H) and mediastinal (window width 400 H, window level 20 H) windows. Right upper lobe (RUL), right lower lobe (RLL), right middle lobe (RML), left upper lobe (LUL), lingula, and left lower lobe (LLL) were reviewed for each patient, accounting for 204 lobes in total.

Statistical analysis was performed by SPSS software for Windows (version 16; IBM Corporation, Armonk, NY, USA). Qualitative analysis was conducted to figure out the frequency of CT scan findings. The results were reported in both the frequency and percentage. Zonal distribution of opacities was analyzed with *t*-test and related *p* values were reported. The bronchiectasis severity was evaluated based on the CT scoring system for cystic fibrosis. The cases were scored from 1 to 3 [21, 22]. Adenopathy station was reviewed based on the International Association for the Study of Lung Cancer (IASLC) lymph node map [23].

## 3. Results

In this study, we reviewed CT scan images of 34 patients with average age of 63 ± 14.54 years. 18 patients (52.9%) were male. All patients were of Iranian origin. Habitual history included smoking in 22 patients (64.7%), opium abuse in 10 patients (29.4%), and alcohol abuse in two patients (5.9%).

The presenting symptoms of lung infection were cough in 31 patients (91.2%) and sputum production in 26 patients (76.5%). Other symptoms included fever (18 patients, 52.9%), dyspnea (18 patients, 52.9%), weight loss (14 patients, 41.2%), chest pain (13 patients, 38.2%), and sweating (9 patients, 26.5%). Purified protein derivative (PPD) skin test for tuberculosis was positive in 9 patients (26.5%). Underlying medical conditions and habitual history are presented in Table 1.

Lymphadenopathy, pleural effusion, and pleural thickening were seen in 15 (44.11%), 7 (20.6%), and 20 (58.8%) patients, respectively. The most common location of adenopathy was right lower paratracheal (12 patients, 35.3%), followed by right hilar (2 patients, 5.9%) and left hilar (2 patients, 5.9%).

**Figure 1:**
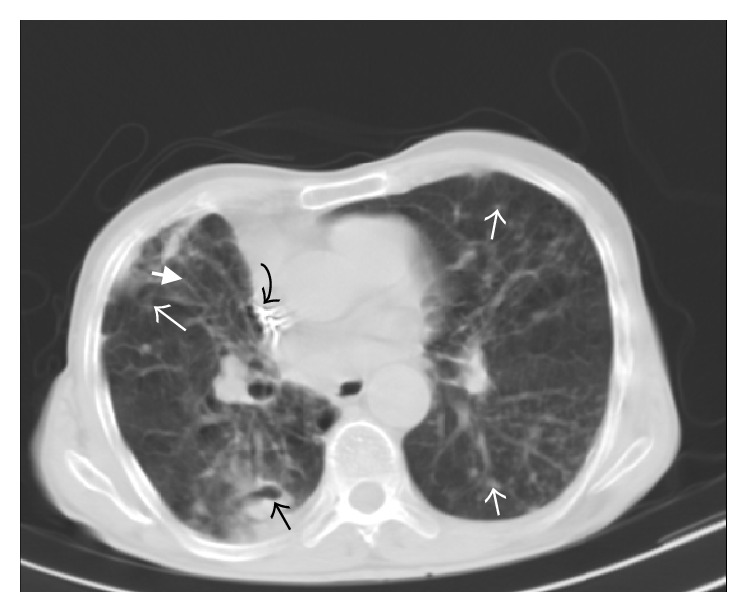
88-year-old male. Spiral CT scan at the level of aortic root (lung window). Cavitary nodule in right lower lobe (black arrow) along with nodular infiltration in left lower lobe, lingula, right lower lobe, and right middle lobe (white arrows). Note also cylindrical bronchiectasis in right middle lobe (thick white arrow). The hyperdense focus in superior vena cava was related to cardiac pacemaker (curved arrow).

Out of 204 evaluated pulmonary lobes, bronchiectasis (Figure 1) was detected in 75 lobes (36.8%) and 29 patients (85.29%) had bronchiectasis in at least one lobe. The most commonly involved lobe was RML (50%), followed by lingula (47%), RUL (41.2%), RLL (32.4%), LLL (32.4), and LUL (17.6%). Most patients had mild bronchiectasis.  Table 2 shows frequency of different grades of bronchiectasis in* M. simiae* pulmonary infection.

**Figure 2:**
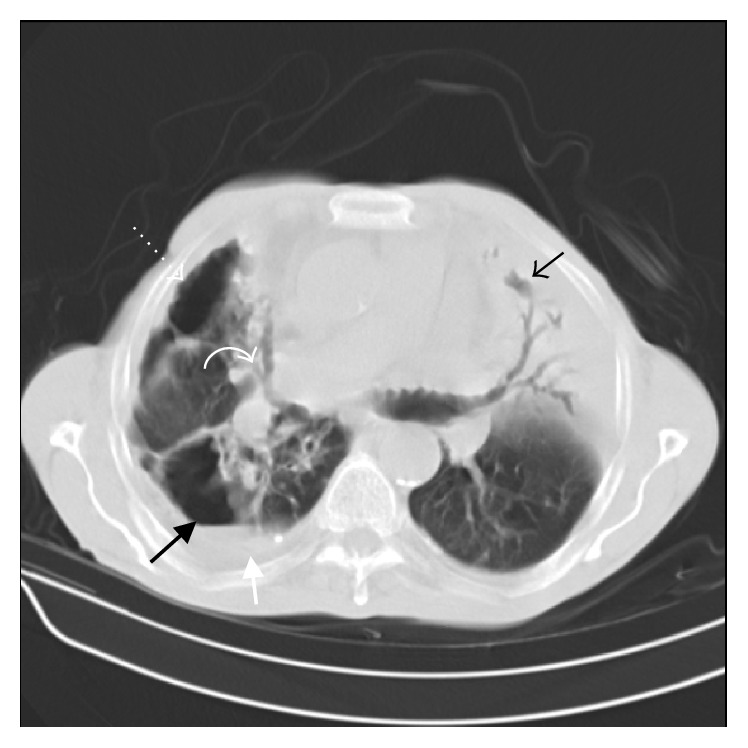
75-year-old male. Spiral CT scan at the level of pulmonary artery (lung window). Note consolidation with air bronchogram in lingula (black arrow) and air-fluid containing cavity in right lower lobe (thick black arrow) adjacent to pleural effusion (thick white arrow). There are also bronchiectasis in right middle lobe (curved arrow) and pneumothorax (dotted arrow).

In this study, all patients had at least one nodule. We categorized nodular opacities according to the size into two groups of under and over one centimeter. Nodules under and over one centimeter were both most common in RLL (58.8% and 26.5%, resp.) (Tables 3 and 4). Cavitation and air-fluid level (Figure 2) were more frequent in nodules over one centimeter (29.4% versus 8.8% for cavitation and 8.8% versus 2.9% for air-fluid level). However, calcification rate did not differ between the two groups.

Tree-in-bud opacities were observed in 30 patients (88.2%). The distribution of tree-in-bud opacities in different lung lobes is as follows: RML (47.1%), LLL (47.1%), RLL (41.2%), RUL (35.3%), LUL (35.3%), and lingula (32.4%).

7 (20.58%) of our patients had masses. In 5 cases (14.70%), the masses demonstrated cavitation. Air-fluid level was observed in 4 patients (11.76%). Masses were most commonly located in RLL (11.8%), RUL (8.8%), and LLL (8.8%).

**Figure 3:**
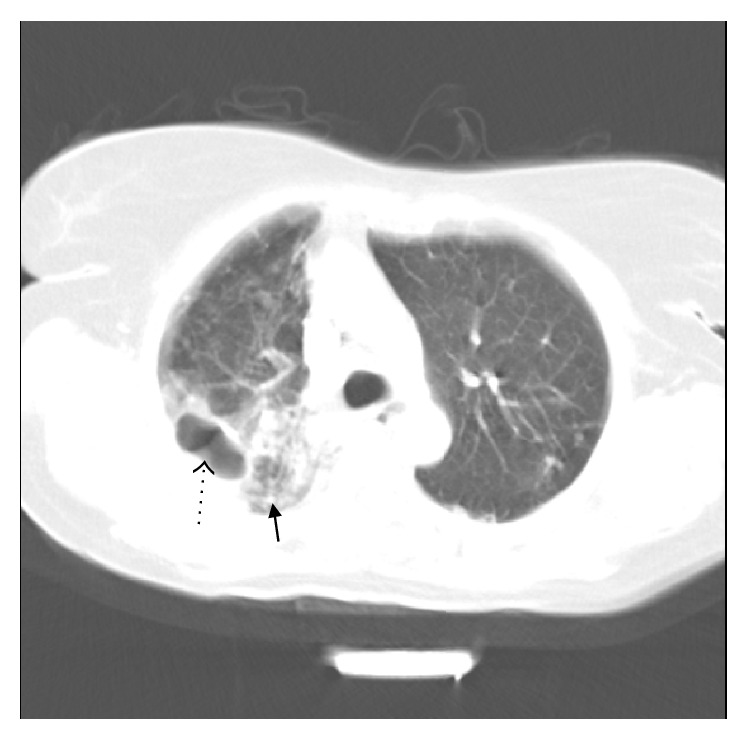
66-year-old female. Spiral CT scan below the level of left pulmonary artery (lung window). There is cavitary consolidation in posterior segment of right upper lobe (dotted arrow) along with adjacent nodular infiltration (thick arrow).

Consolidation (Figures 2 and 3) was observed in 18 (52.94%) cases, 3 (8.82%) of which had cavitation (Figure 3). Consolidation was more prevalent in RUL (20.1%), RML (11.8%), and RLL (11.8%).

Lobar fibrosis and volume loss were seen in 23 cases (67.6%). The locations were RUL (55.9%), RLL (8.8%), LUL (5.9%), and lingula (2.9%). Bulla/Bleb was detected in 8 patients (23.5%). Emphysema, chest wall deformity, and pneumothorax were seen in 14 (41.2%), 18 (52.9%), and 2 (5.9%) patients, respectively.

Although there were some differences in the distribution of CT scan findings in various lung lobes, based on *p* values, none of these differences were statistically significant.

## 4. Discussion

There are very few studies evaluating CT scan findings in patients with* M. simiae* pulmonary infection. The patients' demographic factors and underlying medical conditions were consistent with the previous Iranian study on* M. simiae* pulmonary infection [11]. Maoz et al.'s study has also revealed that smoking, diabetes, cardiac disease, malignancy, and chronic obstructive pulmonary disease have been associated with higher incidence of* M. simiae* infection [24], although the rate of malignancy was not so much high in our study (2.9%).

The most common findings in our study were nodular lesions (100%) and bronchiectasis (85.29%). Other studies have also found almost similar results. Baghaei et al. [11] have reported that patients most commonly had nodular lesions (100%), cavitation (88.5%), and bronchiectasis (84.6%). Christensen et al. [18, 25] stated that ill-defined nodules without cavity and bronchiectasis were the most common CT findings. Consistent with our study, Song et al. [26] found bronchiectasis in 98% and cavitation in 36% of patients. In previous studies, generally, CT scan findings were reported for NTM infections altogether rather than describing manifestations of one specific type. Shitrit et al. made a comparison between two species of mycobacteria and found lower rates of cavitation in* M. simiae* than* M. kansasii* (3% versus 57%, *p* value = 0.001) [14].

Lymphadenopathy was found in 46.1% of patients in our study. Lee et al. have reported similar rates around 33.3% [25]. In contrast to our findings, Banks et al. have detected lymphadenopathy in 3% of the patients [14, 27]. Also, 20.6% of our patients had pleural effusion while in other studies this figure varied from 4.16% to 16% [14, 25].

The NTM infection patterns can be quite different between immunocompetent and immunocompromised patients. Due to paucity of immunocompromised patients in our study, we can consider our patients as immunocompetent. In immunocompetent patients, two major patterns have been described for NTM infection: an upper lobe cavitary pattern and a nodular bronchiectatic pattern [28–33]. In our study, nodular bronchiectatic form without any significant zonal distribution was more common.

The previous TB infection rate was high in our study (41.2%). In the available literature, it is reported that history of pulmonary diseases including TB is more common in NTM infections [34, 35]. Shitrit et al. [14] support these findings and declare that the rate of previous pulmonary disease in* M. simiae* group is higher than* M. kansasii* patients.

Rates of scar and volume loss in different studies have been reported from 11% to 36% [26, 34] but this rate in our study was even higher and reached 67.6%. The cause of high rate of scar and volume loss in our study is not clear and should be investigated in further researches.

Our study's main limitations were lack of second group for comparison, its retrospective type, and limited number of patients. In future studies, presence of second group (control or infections of other types) can help obtaining more solid conclusions. In conclusion, nodular lesions and bronchiectasis are the most frequent features in CT scan findings of* M. simiae* pulmonary infection.

## Figures and Tables

**Table 1:** Underlying conditions in medical and habitual history (CRF: chronic renal failure, COPD: chronic obstructive pulmonary disease).

Predisposing factors in medical and habitual history	Frequency
Smoking	22 (64.7%)
Opium consumption	10 (29.4%)
Alcohol abuse	2 (5.9%)
Malignancy	1 (2.9%)
CRF	1 (2.9%)
Diabetes mellitus	12 (35.3%)
Immunodeficiency	1 (2.9%)
TB history	14 (41.2%)
Cardiac disease	13 (38.2%)
Chest wall deformity	12 (35.3%)
COPD	6 (17.6%)
Cystic fibrosis	7 (20.6%)

**Table 2:** Frequency of different grades of bronchiectasis in *M. simiae *pulmonary infection.

Bronchiectasis grade	Frequency	*p* value
I	25 (73.5%)	0.011
II	8 (23.5%)	
III	1 (2.9%)	

**Table 3:** Frequency of locations of nodules under one centimeter.

Location	Frequency	*p* value
Right upper lobe	16 (47.1%)	0.447
Right middle lobe	18 (52.9%)	
Right lower lobe	20 (58.8)	
Left upper lobe	17 (50%)	
Lingula	12 (35.3%)	
Left lower lobe	19 (55.9%)	

**Table 4:** Frequency of locations of nodules over one centimeter.

Location	Frequency	*p* value
Right upper lobe	5 (14.7%)	0.392
Right middle lobe	7 (20.6%)	
Right lower lobe	9 (26.5%)	
Left upper lobe	5 (14.7%)	
Lingula	6 (17.6%)	
Left lower lobe	6 (17.6%)	
